# Phylogeographic Structure in Benthic Marine Invertebrates of the Southeast Pacific Coast of Chile with Differing Dispersal Potential

**DOI:** 10.1371/journal.pone.0088613

**Published:** 2014-02-19

**Authors:** Pilar A. Haye, Nicolás I. Segovia, Natalia C. Muñoz-Herrera, Francisca E. Gálvez, Andrea Martínez, Andrés Meynard, María C. Pardo-Gandarillas, Elie Poulin, Sylvain Faugeron

**Affiliations:** 1 Laboratorio de Diversidad Molecular, Departamento de Biología Marina, Facultad de Ciencias del Mar, Universidad Católica del Norte & Centro de Estudios Avanzados en Zonas Áridas (CEAZA), Coquimbo, Chile; 2 Interdisciplinary Center for Aquaculture Research (INCAR), Universidad de Concepción, Concepción, Chile; 3 Instituto de Ecología y Biodiversidad, Departamento de Ciencias Ecológicas, Universidad de Chile, Santiago, Chile; 4 Centro de Conservación Marina, Facultad de Ciencias Biológicas, Pontificia Universidad Católica de Chile, Santiago, Chile; Technical University of Denmark, Denmark

## Abstract

The role of dispersal potential on phylogeographic structure, evidenced by the degree of genetic structure and the presence of coincident genetic and biogeographic breaks, was evaluated in a macrogeographic comparative approach along the north-central coast of Chile, across the biogeographic transition zone at 30°S. Using 2,217 partial sequences of the mitochondrial Cytochrome Oxidase I gene of eight benthic invertebrate species along ca. 2,600 km of coast, we contrasted dispersal potential with genetic structure and determined the concordance between genetic divergence between biogeographic regions and the biogeographic transition zone at 30°S. Genetic diversity and differentiation highly differed between species with high and low dispersal potential. Dispersal potential, sometimes together with biogeographic region, was the factor that best explained the genetic structure of the eight species. The three low dispersal species, and one species assigned to the high dispersal category, had a phylogeographic discontinuity coincident with the biogeographic transition zone at 30°S. Furthermore, coalescent analyses based on the isolation-with-migration model validate that the split between biogeographic regions north and south of 30°S has a historic origin. The signatures of the historic break in high dispersers is parsimoniously explained by the homogenizing effects of gene flow that have erased the genetic signatures, if ever existed, in high dispersers. Of the four species with structure across the break, only two had significant albeit very low levels of asymmetric migration across the transition zone. Historic processes have led to the current biogeographic and phylogeographic structure of marine species with limited dispersal along the north-central coast of Chile, with a strong lasting impact in their genetic structure.

## Introduction

It has long been established that the geographical structure of the genetic diversity of benthic species is strongly shaped by life history traits. Among the main life history attributes, mode of larval development, duration and characteristics of larval life, have been highlighted as features that influence the maximum distance of dispersal in many benthic marine species [1]–[3]. Similarly it has been recognized that dispersal potential in terms of the pelagic larval duration (PLD) is an important determinant of the genetic structure of benthic marine species. Many of these taxa have planktonic larvae that can spend up to several months and even over a year in the water column; others spend only hours or few days in the water column, theoretically preventing long distance dispersal and eventually reducing population connectivity [4].

In the recent years however, the correlation between pelagic larval duration (PLD) and the spatial scale of genetic differentiation has been repeatedly questioned by meta-analyses and comparative studies of benthic and coastal marine species [5]–[10]. These studies have consistently shown a low to null correlation, suggesting that PLD is a poor predictor of connectivity between marine populations. This conclusion, however, has to be considered with caution, as it seems to be largely influenced by the kind of markers, sampling scheme and genetic parameters used to determine the genetic structure [11]. Alternative determinants of spatial genetic structure have been proposed, such as vicariant effects of historic patterns of oceanic circulation (e.g. Plio–Pleistocene), physical barriers and contemporary restrictions to dispersal, including modern patterns of oceanographic circulation and the distribution of suitable habitat ([12] and references therein). There are also mechanisms that enhance connectivity such as anthropogenic transport (e.g. [13]–[14]) or rafting on floating objects [15]–[16], allowing connectivity between distant geographic areas.

Among the different alternatives, biogeographic transitions have been considered as ideal situations to study the historical and contemporary processes that affect the distribution and abundance of species. Indeed, depending on the species ability to disperse and survive in different environmental conditions, biogeographic breaks often reflect species range limits [17]. In fact, three main patterns are observed: a) species that end their geographic range within this limited geographic area, b) species that cross the transitional zone, but their population density and structure changes dramatically, leading to major changes in dominance within local communities, and c) species that cross the boundary and do not seem to “perceive” any discontinuity as reflected in their homogeneous abundance. An increasing number of reports are evidencing strong phylogeographic structure associated to biogeographic breaks in these latter species, allowing the inference of the origin and the persistence of the biogeographic structure (e.g. [18]–[20]). For example, the southeast Australian coast bears a major biogeographic transition between two different oceanographic conditions and coincides with the Pleistocene land-bridge formation between Australia and Tasmania. This transition is therefore the result of both a historical and a contemporary barrier to dispersal, and coincides with deep phylogeographic differentiation and/or restricted gene flow [21]–[24]. The different studies conducted so far suggest that both the historical barrier and the contemporary oceanic circulation have caused genetic lineages to diverge and remain geographically separated [25]. In this region, potential dispersal inferred from larval type is less correlated with the presence of a phylogeographic break than habitat specificity [12]. However, other studies are consistent with the hypothesis that dispersal potential is determinant to the presence or location of phylogeographic breaks [18], [26]. Pelc et al. [26] analyzed datasets of marine fauna of the southwest and southeast coast of the USA to compare concordance among phylogeographic and biogeographic breaks. Dispersal potential appeared to be a strong determinant of the magnitude and position of phylogeographic discontinuities, associated to biogeographic breaks. These results strongly emphasize that phylogeographic breaks of species with low dispersal potential reflect primarily historical processes whereas those of species with high dispersal potential reflect more recent or current gene flow restrictions [26]–[29]. The rational behind these patterns is that phylogeographic structure can persist many generations for species with restricted gene flow, whereas high gene flow species can bear a phylogeographic break only if a local barrier is currently restricting dispersal, whatever the history of the barrier [30].

This study further tests these predictions by undertaking a comparative phylogeographic analysis across a biogeographic boundary along the coast of Chile. The lineal coast of Chile, north of 42°S, encompasses approximately 2,600 km with a natural temperature gradient associated to latitude, but with neither apparent geographic barriers to dispersal nor any sharp environmental discontinuity. The cold and rich nutrient Humboldt Current influences the coastal area between 18 and 42°S, with upwelling as the dominant feature influencing coastal communities [31]. In spite of this, a marine biogeographic break has been reported at 30°S based on the distribution ranges of most benthic species (for review see [31]–[32]). The origins of this marine discontinuity as well as the environmental factors that ensure its persistence are largely unknown. One question that is still open is if there is a present-day disruption in the ecological characteristics of the coastal ecosystems, or if the biogeographic break results from some historic vicariant or disruptive event that no longer persist. Three phylogeographic studies conducted so far have evidenced a strong phylogeographic break coinciding with the 30°–33°S region: the kelp *Lessonia nigrescens* [33], the red alga *Mazzaella laminarioides* [34], and the brooding muricid gastropod *Acanthina monodon* [35], suggesting the existence of an old divergence between lineages north and south of 30°S. Three other species, the muricid gastropod *Concholepas concholepas*, and the squat lobster *Pleuroncondes monodon* and the cephalopod *Dosidicus gigas,* did not show genetic structure at a macrogeographic scale across 30°S [36]–[38]. Taxa with low dispersal that lack the break correspond to species that are likely to disperse by alternative means, the algal-dwelling genus *Limnoria* by rafting on macroalgae [39] and the biofouling tunicate *Pyura chilensis* on boat and ship hulls [40]. Even though these studies have important differences in the sampling effort, the dispersal potential of these species seems tightly related to the magnitude of phylogeographic breaks and their concordance with the biogeographic transition at ca. 30°S. *Lessonia nigrescens* and *A. monodon* have planktonic dispersive stages that last a few hours to a few days, whereas *C. concholepas*, *J. cirratus*, *P. monodon* and *D. gigas* can spend weeks to several months in the water column. Thus, these studies strongly suggest that the biogeographic break is the result of a past discontinuity that is currently not perceivable in the macrogeographic genetic structure of species with high dispersal potential.

The current study aims at contributing to the knowledge of phylogeographic patterns of marine benthic organisms along the Chilean coast under the influence of the Humboldt Current focusing on eight species with high and low dispersal potential. More broadly, the role of dispersal potential on the population genetic diversity and differentiation is addressed. We predict that species with low dispersal potential will have greater genetic structure, lower genetic diversity than high dispersers and the presence of a historic phylogeographic break around 30°S, coincident with the biogeographic transition zone.

The species chosen for this study are all very abundant in the intertidal of the Chilean coast in the two biogeographic regions at the vicinity of 30°S, and none of them has commercial value. For categorization purposes, the average number of days that larvae spend in the water column was used as a proxy of PLD (see Materials and Methods) with reflects an upper bound of dispersal rather than a mean dispersal time [10]. Species that do not have free-living larvae or with PLD in the water column of less than two weeks were considered as low dispersers while those whose larvae spend 15 or more days in the water column were considered as high dispersers. Based on this, five of the model species fell in the long dispersal potential category. These include two species of the Order Decapoda (Infraorder Anomura), *Emerita analoga* (Stimpson) (Hippidae) and *Petrolisthes violaceus* Stimpson (Porcellanidae); two starfishes of the class Asteroidea (order Forcipulatida), *Heliaster helianthus* (Lamarck) (Heliasteridae) and *Stichaster striatus* Müller and Troschel (Asteriidae); and the sea urchin *Tetrapygus niger* (Molina) (Echinoidea, Arbaciidae). The remaining three species were considered as having low dispersal potential: the peracarid *Orchestoidea tuberculata* Nicolet (Amphipoda, Talitroidea) that broods its embryos, and the gastropods *Scurria scurra* (Lesson) (Patellogastropoda, Lottidae) and *Tegula atra* (Lesson) (Vestigastropoda, Trochoidea, Turbinidae, Tegulinae) (see Materials & Methods section; Table 1).

**Table 1:** Species analyzed and the corresponding depth range in meters that they inhabit, and dispersal potential (DP) as number of days that larvae spend in the water column.

Phylum	Species	Depth range	DP
Crustacea	*Emerita analoga*	0–3	>90
	*Orchestoidea tuberculata*	0	*0
	*Petrolisthes violaceus*	0–3	25
Mollusca	*Scurria scurra*	0–20	*7–10
	*Tegula atra*	0–9	*6–7
Echinodermata	*Heliaster helianthus*	0–10	>60
	*Stichaster striatus*	1–80	38
	*Tetrapygus niger*	0–40	>90

*Considered as low dispersers in this study.

## Results

**Figure 1:**
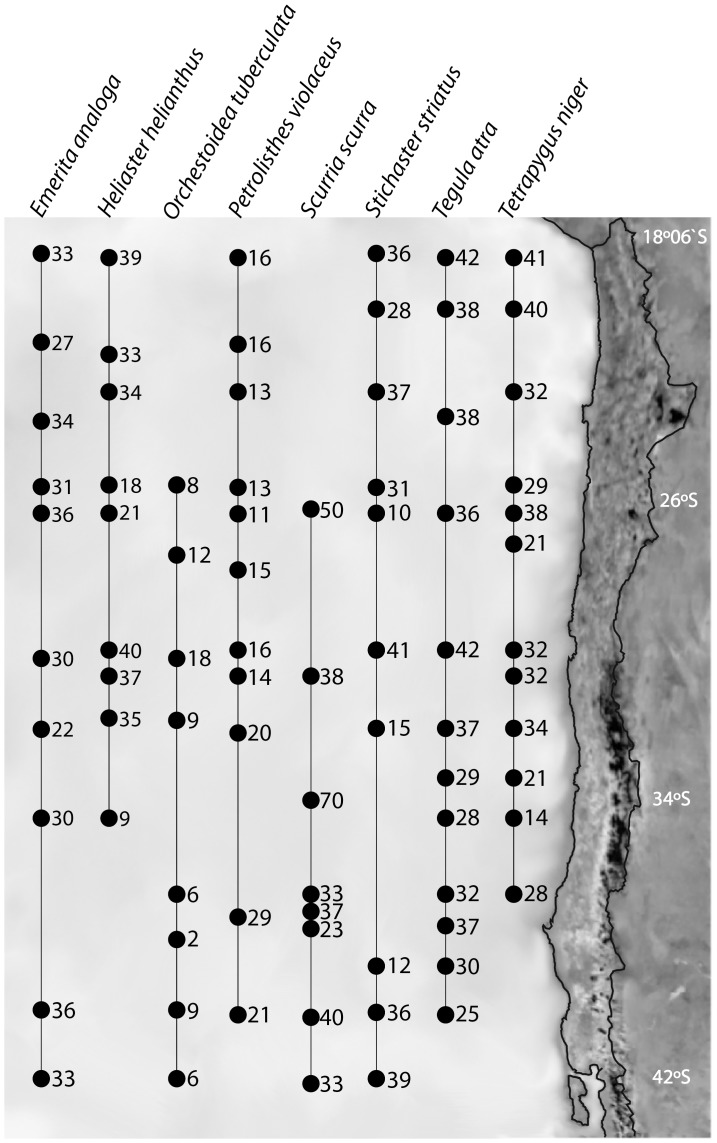
Map of the study area along the coast of Chile indicating the sampling sites and the sample size per site for each of the analyzed species.

COI gene sequences were obtained from 2,217 individuals of eight species (Table 2), covering at least 16 and up to 23 degrees of latitude along the coast of Chile (Fig. 1). Sequences were deposited in the GenBank database with the following accession numbers: *Emerita analoga* JX130229 - JX130344; *Heliaster helianthus* JX130054 - JX130098; *Orchestoidea tuberculata* JX129929 - JX129953; *Petrolisthes violaceus* KC543356 - KC543409; *Scurria scurra* JX130193 - JX130212; *Stichaster striatus* JX129954 - JX130053; *Tegula atra* JX130156 - JX130192; *Tetrapygus niger* JX130099 - JX130155.

**Table 2:** Latitudinal range analyzed in degrees for each species (Lat); number of localities (NL); number of sequences analyzed per species (NS); length of final truncated alignment (LA); percentage of variable sites (%V); average haplotype (h) and nucleotide (π) diversities; S_nn_; global Φ_ST_, and Mantel correlation coefficient (MCC).

Species	Lat	NL	NS	LA	%V	h	π	S_nn_	Φ_ST_	MCC
*Emerita analoga*	23	10	312	606	16.17	0.7643	0.0042	0.1429	−0.001	−0.317
*Heliaster helianthus*	16	9	266	630	6.35	0.8654	0.0034	0.1464	0.0093	0.139
*Orchestoidea tuberculata*	16	8	70	633	23.22	0.5744	0.0021	**0.9310**	**0.6175**	**0.528**
*Petrolisthes violaceus*	21	11	184	591	8.12	0.7849	0.0024	0.1320	0.0231	0.056
*Scurria scurra*	16	8	324	672	2.66	0.3620	0.0005	**0.3261**	**0.7976**	**0.701**
*Stichaster striatus*	23	10	285	609	14.12	0.8253	0.0063	**0.1883**	**0.2590**	**0.834**
*Tegula atra*	21	12	414	663	5.28	0.4392	0.0008	**0.1336**	**0.3625**	**0.682**
*Tetrapygus niger*	17	12	362	546	8.43	0.8201	0.0035	0.0837	−0.087	−0.172
**Average**	18.6	10	277	619	10.54	0.6795	0.0029	0.2605	0.2476	0.306

Significant values are in bold.

**Figure 2:**
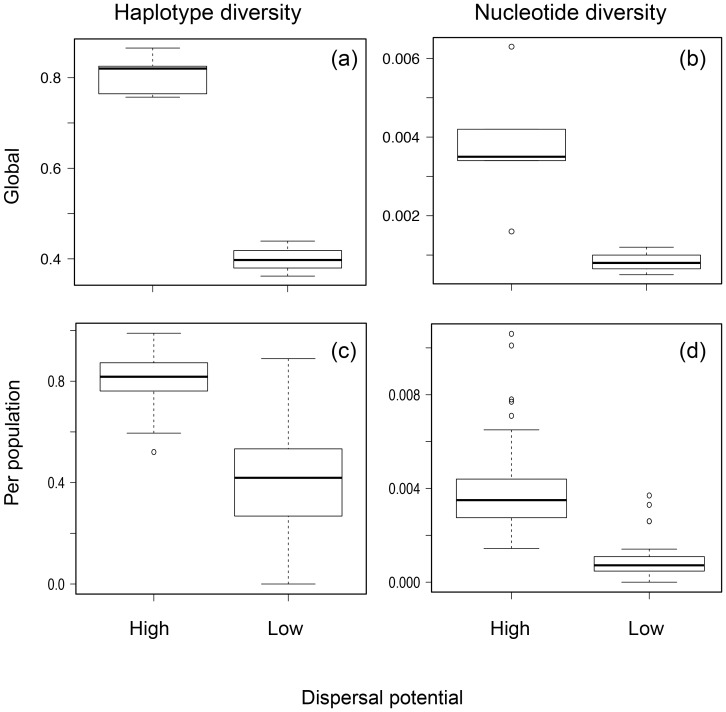
Box-plots of global (a and b) and per population (c and d) haplotype (a and c) and nucleotide (b and d) diversities for high and low dispersers.

All truncated alignments lacked indels and their final length varied from 546 nucleotides in *T. niger* to 672 in *S. scurra*. Variable sites varied between 2.7% in *S. scurra* to 23.2% in *O. tuberculata*, with an average of 10.5% (Table 2). Most substitutions corresponded to transitions; only *O. tuberculata* exhibited average transversions greater than zero, with 37.5% of substitutions corresponding to transversions. Percentage of variable sites, as well as haplotype and nucleotide diversities, were in general high in high PLD species while low dispersers showed lower values (Table 2). Generalized Linear Mixed Models (GLMMs) consistently revealed that dispersal potential was the variable that best explained genetic diversity patterns (Fig. 2; Table 3). In the case of nucleotide diversity, biogeographic region (north or south of 30°S) was also a significant explanatory variable, as well as its interaction with dispersal potential (Table 3).

**Table 3:** GLMMs performed to compare genetic structure in species with high and low dispersal potential and biogeographic regions (north and south of 30°S), with respect to haplotype diversity, nucleotide diversity, number of substitutions, and genetic differentiation (global Φ_ST_).

GLMM	Estimate	Standard error	t-value		*P*
Haplotype Diversity: AIC = −35.33; *df* = 7					
Intercept	0.804	0.029	29.259		**0.001**
DP	−0.395	0.054	−7.590		**0.001**
BR	0.016	0.046	0.361		0.712
DP x BR	−0.016	0.075	−0.227		0.816
Nucleotide Diversity: AIC = −750.4; *df* = 7					
Intercept	0.004	0.001	5.370		**0.001**
DP	−0.002	0.001	−3.144		**0.008**
BR	0.001	0.000	2.714		**0.007**
DP x BR	−0.001	0.001	−2.339		**0.019**
Number of Substitutions: AIC = −505.3; *df* = 7					
Intercept	15.219	3.034	5.306		**0.001**
DP	−10.469	3.416	−3.221		**0.008**
BR	1.935	1.581	1.284		0.190
DP x BR	−3.155	2.588	−1.261		0.199
Genetic differentiation (pairwise Φ_ST_): AIC = −518.5; *df* = 7					
Intercept	0.057	0.027	2.216		**0.043**
DP	0.661	0.235	2.827		**0.028**
BD	−0.036	0.014	−2.925		**0.004**
DP x BD	−0.319	0.024	−14.163		**0.001**

Species were nested to dispersal in the models. For each model the AIC value is shown and the variance estimate of fixed effects (Estimate), standard error, degrees of freedom (*df*), t-value ratio and *P* values of the intercept and of the contributions of dispersal potential (DP), biogeographic region (BR) (for genetic diversities and number of substitutions) or biogeographic differentiation (BD) (for genetic differentiation), and the interaction of DP x BR and DP x BD for diversities and differentiation, respectively.

Significant values are in bold (*P*<0.05).

**Figure 3:**
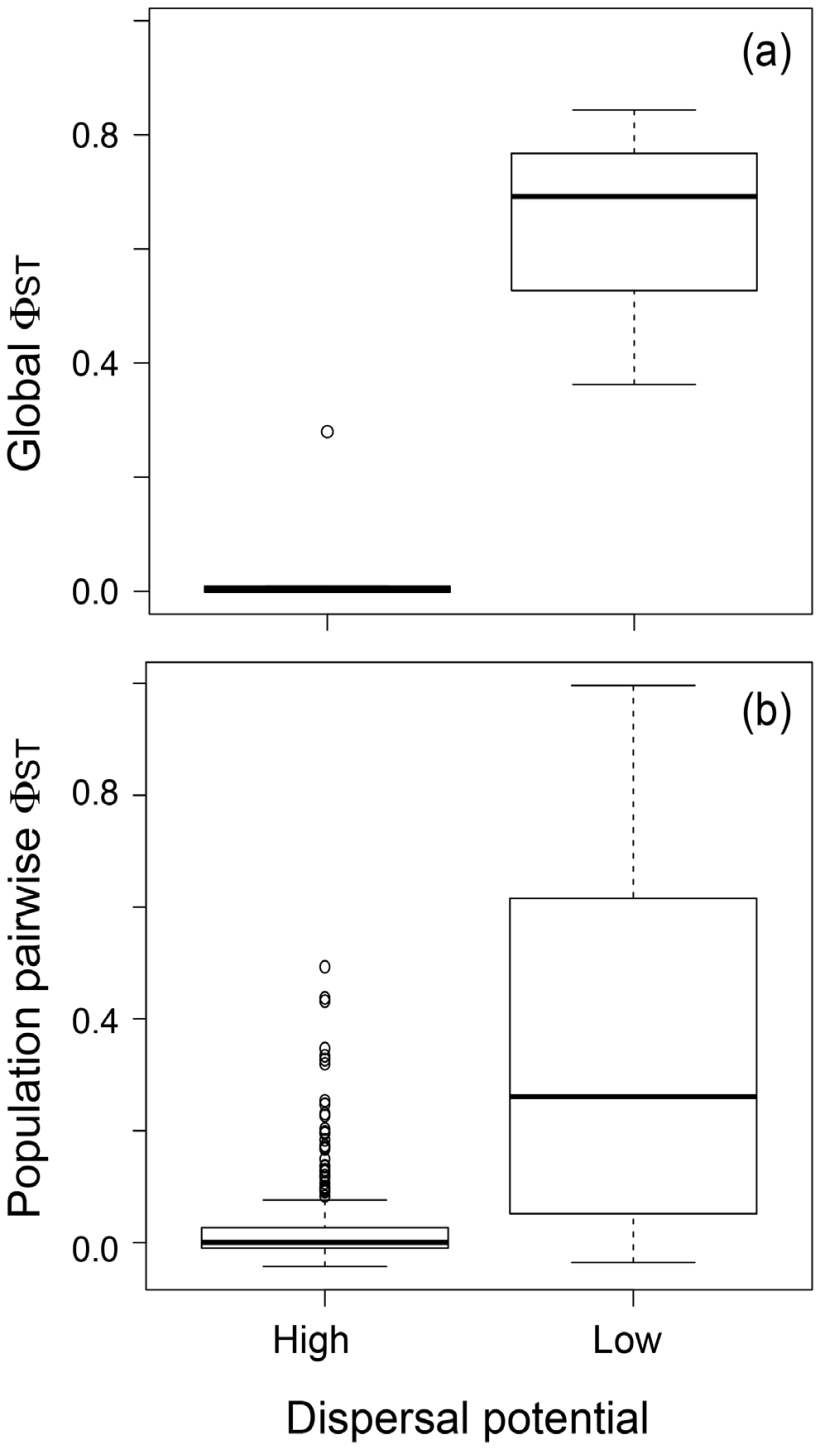
Box-plots of global (a) and average population-pairwise (b) Φ_ST_ for high and low dispersers.

S_nn_, average pairwise Φ_ST_, and Mantel’s correlation coefficient for isolation by distance were significant in all low dispersers and for the starfish *S. striatus*; the other species showed non-significant values (Table 2). The low dispersers *O. tuberculata* and *S. scurra* had the highest S_nn_ values, 0.93 and 0.33, respectively, indicating that most individuals that were genetically similar occurred in the same locality. Likewise, the highest Φ_ST_ values were found in *S. scurra* and *O. tuberculata* (Table 2), and overall, low dispersers showed greater genetic differentiation than high dispersers (Fig. 3). Mantel’s correlation coefficients were highest for the three low dispersers, which also showed significant isolation by distance in each biogeographic region. Isolation by distance was also significant in *S. striatus*, however analyses per biogeographic region indicated a significant Mantel’s correlation coefficient only in the southern biogeographic region. Similarly *S. striatus* had significant Φ_ST_ only in the southern region, while for the three low dispersers Φ_ST_ values were significant in both biogeographic regions (data not shown). The genetic structure detected for *S. striatus* was weaker than on the three low dispersers.

Overall, GLMMs indicated that pairwise Φ_ST_ values were significantly associated with the interaction of dispersal potential and biogeographic differentiation (i.e., genetic differentiation among or within biogeographic regions; Table 3). When excluding separately the brooder *O. tuberculata* and the starfish *S. striatus*, GLMMs of genetic diversity and differentiation yielded overall similar results, giving support to the robustness of the detected patterns. The exclusion of *O. tuberculata* was aimed at testing the effects of non-pelagic dispersal (see [5]) on the results. If its removal from analyses resulted in a non-significant relationship between dispersal potential and genetic differentiation, then dispersal potential would poorly correlate with genetic differentiation of species with free larval development, i.e. broadcasters. On the opposite, since *S. striatus* had more genetic structure than expected given the dispersal potential that was assigned based on a congener, we evaluated the effects of this species on the GLMM results. However, none of the exclusions led to a change in the association between dispersal potential and measures of genetic diversity and differentiation (data not shown), suggesting that differences detected between low and high dispersers were not biased by the inclusion of *O. tuberculata* and *S. striatus*.

AMOVA performed grouping populations by biogeographic region indicated that low dispersers had the highest percentages of variation between biogeographic regions, while high dispersers had the highest percentages of variation within populations (Table 4). *Tegula atra* and *S. striatus* showed a very similar pattern; although there was greater structure in *T. atra* both species had a significant and high portion of the total variance within populations (63.7% and 74.1%, respectively), and a considerable amount explained by biogeographic region (29.6 and 23.8%, respectively, although non-significant for *S. striatus*). Overall, GLMMs revealed significant differences between high and low dispersers for each of the AMOVA’s Φ-statistics (Table 5).

**Table 4:** AMOVA for all species.

Species	Among Groups (N and S of 30°S)	Among populations within groups	Within populations
*Emerita analoga*	−0.29	0.09	100.2
*Heliaster helianthus*	−0.62	1.54	99.07
*Orchestoidea tuberculata*	**36.09**	**48.28**	**15.64**
*Petrolisthes violaceus*	0.42	1.89	97.69
*Scurria scurra*	**69.15**	**10.60**	**20.24**
*Stichaster striatus*	**23.78**	2.11	**74.1**
*Tegula atra*	**29.58**	**6.67**	**63.75**
*Tetrapygus niger*	−0.03	−0.84	100.87

Percentage of variation explained among groups north and south of 30°S, among populations within groups, and within populations are shown.

Values in bold indicate association to significant AMOVA’s Φ-statistics.

**Table 5:** GLMMs performed to compare species with high and low dispersal potential (DP) in AMOVA’s F statistics among groups (Φ*_CT_*), among populations within groups (Φ*_SC_*), and within populations (Φ*_ST_*).

GLMM	Estimate		Standard error	t-value	*P*
**Among Groups (Φ** ***_CT_*** **): AIC = 7.388; ** ***df*** ** = 3**					
**Intercept**	0.050		0.171	0.295	0.497
**DP**	0.435		0.251	1.739	**0.017**
**Among populations within groups (Φ** ***_SC_*** **): AIC = 8.009; ** ***df*** ** = 3**					
**Intercept**	0.012		0.180	0.064	0.874
**DP**	0.386		0.264	1.463	**0.025**
**Within Populations (Φ** ***_ST_*** **): AIC = 7.259; ** ***df*** ** = 3**					
**Intercept**	0.045		0.169	0.264	0.540
**DP**	0.588		0.248	2.372	**0.008**

Species were nested to dispersal in the model. For each model the AIC value, and the variance estimate of fixed effects (Estimate), standard error, degrees of freedom (*df*), t-value ratio and *P* values of the intercept are shown.

Significant values are in bold (*P*<0.05).

**Figure 4:**
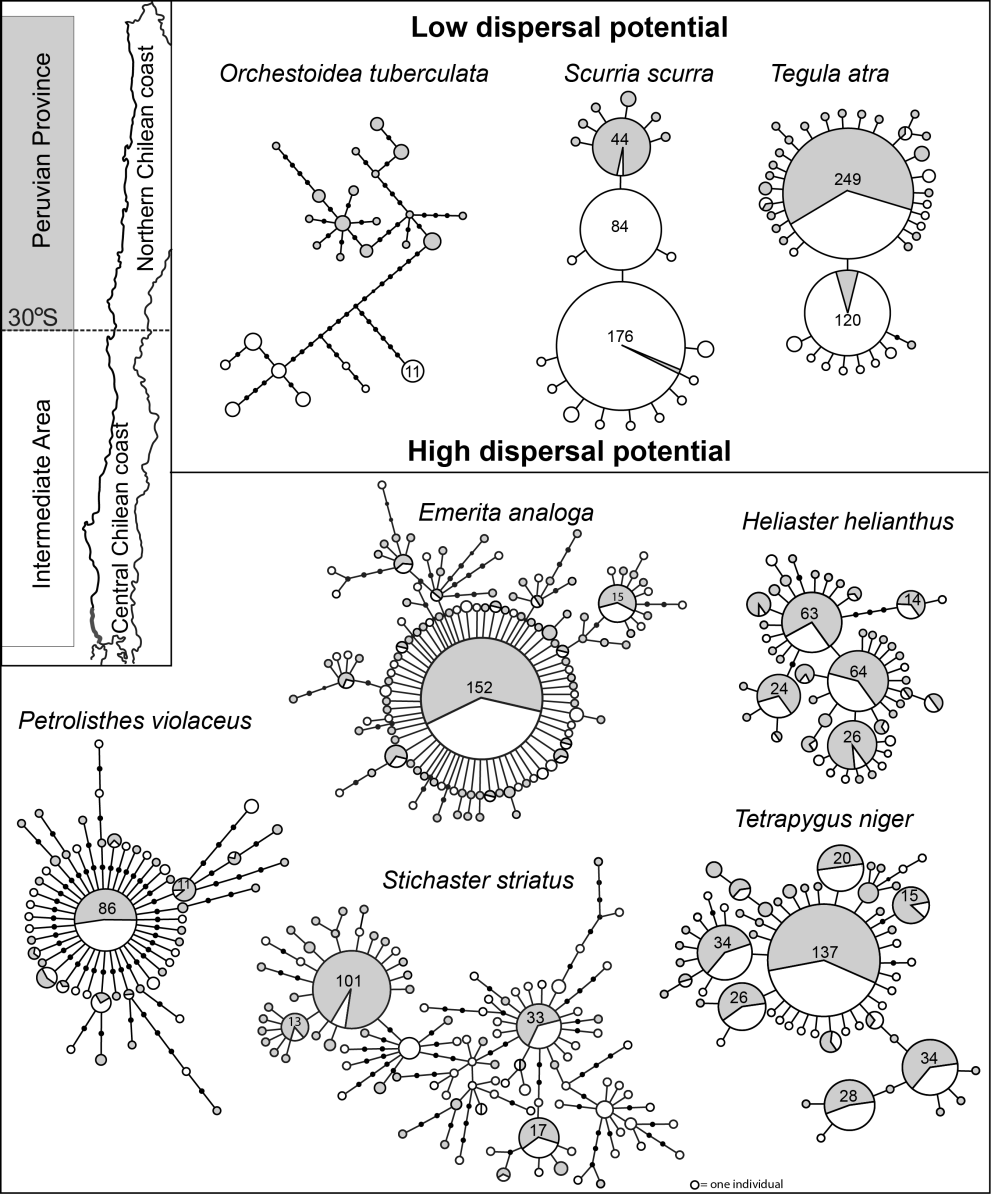
Median joining haplotype networks of eight species analyzed of the Chilean coast. Insert with map details the biogeographic regions north and south of 30°S in the analyzed coast section and shades associated with each of them that were used to mark the geographic location of haplotypes. All haplotype networks are standardized in such way that small circles represent haplotypes present in one individual. Mutational steps (lines between circles) were kept the same length excepting for the network of *Emerita analoga* where mutational steps arising from the most common haplotype were kept to the minimum length to allow accommodating all derived haplotypes.

Mitochondrial DNA diversity was high (Fig. 4). Most species had a most frequent haplotype shared between biogeographic regions. The exception was the amphipod *O. tuberculata* that showed divergent haplogroups separated by up to 18 mutational steps and lacked shared haplotypes. For the other seven species, the percentage of shared haplotypes between biogeographic regions ranged from 13.5% in *T. atra* to 37.8% in *H. helianthus*; the maximum number of mutational steps was 5 in *S. striatus* and most haplotypes were separated by one mutational step (Fig. 4). In general low dispersers have less shared haplotypes and more private haplotypes in each biogeographic region. Even though the most frequent shared haplotypes were rarely unique of individuals of one biogeographic region, low dispersal species had greater differences on the haplotype frequencies between regions. For example, the most common haplotype of *T. atra* was most often encountered on individuals north of 30°S, and the second most common haplotype was largely composed of individuals from the south of 30°S.

SAMOVA analyses were undertaken on species that displayed significant genetic structure (Table 2) (Fig. 4), i.e. all low dispersers and *S. striatus*, to better characterize the spatial genetic structure. The optimal *k,* that defines the most probable number of geographic subdivisions, ranged between 2 for *S. striatus* and *T. atra*, indicating a low substructure, to 17 for the brooder *O. tuberculata* (Table 6), revealing a strong subdivision of the genetic diversity. For *O. tuberculata* small sample size could lead to overestimation of divergence, but haplotype networks also validate that SAMOVA results reflect degree of divergence between localities. The limpet *S. scurra* had *k = *4 (Table 6). For both *O. tuberculata* and *S. scurra*, when *k* is set to 2, individuals were grouped to the north and south of 30°S (the locality at 31.8°S belonged to the southern group; Table 6). On the other hand, the two groups formed for *S. striatus* and of *T. atra* were located north and south of 31.8°S. *Tegula atra* showed a slightly peculiar pattern: the locality at 34.4°S was included in the northern group while the northern locality at 31.8°S was part of the southern group. In Table 6 this is expressed as a range of latitudes where the discontinuity occurs.

**Table 6:** SAMOVA of species with spatial genetic structure.

Species	Best *k*	F*_CT_*	*P*	*k* (2) F*_CT_*	*k* (2) *P*	Lat *k(*2)
*Orchestoidea* *tuberculata*	17	0.92960	**0.00000**	0.56871	**0.00000**	30°S
*Scurria* *scurra*	4	0.72027	**0.03812**	0.69155	0.13392	30°S
*Stichaster* *striatus*	2	0.24874	**0.00489**	–	–	31.8°S
*Tegula atra*	2	0.35528	**0.00196**	–	–	31.8–34.4°S

Best *k* corresponds to the detected optimal number of populations, the F*_CT_* value of optimal *k* and associated probability (*P*) are shown. For the two taxa that have optimal *k* >2, the corresponding F*_CT_* is also shown (*k* (2) F*_CT_*), as well as the associated *p*-value (*k* (2) *P*), and the latitude that limits the two groups (Lat *k* (2)).

Significant values are in bold.

**Figure 5:**
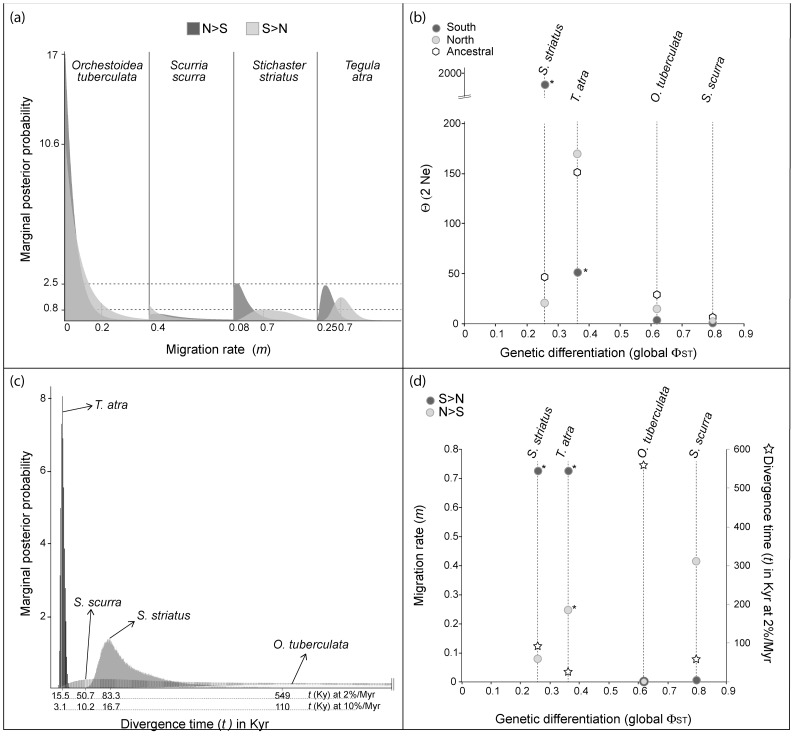
Isolation-with-migration analysis of four species with significant structure across the 30°S biogeographic break. (a) Marginal posterior probability distribution of migration rate estimates in each direction for each species. (b) Estimated Θ (2 Ne) values for each biogeographic region (north and south) as well as the ancestral Θ against genetic differentiation (global Φ_ST_). Asterisks denote estimated Θ 95% HPD that do not overlap with either ancestral Θ or the estimated Θ in the other biogeographic region. (c) Marginal posterior probability distributions of divergence time (*t*) estimates expressed in thousands of years (Kyr). Divergence time estimates were scaled using 2% and 10% per million year substitution rates. (d) Highest probability value of estimated migration rates (*m*) in each direction, and divergence times (*t*) expressed in thousands of years (Kyr) scaled using 2% per million year substitution rate, versus genetic differentiation (global Φ_ST_). Asterisks indicate significant values based on the likelihood ratio test.

The coalescent approach of isolation-with-migration implemented in IMa2 revealed overall low levels of gene flow across the 30°S transition zone in all taxa that displayed significant genetic differentiation between biogeographic regions (north and south of 30°S) (Fig. 5A). In general, the model predicted a most likely pattern of asymmetric migration with greater migration from south to north (Table 7). Similarly, effective population sizes, inferred from Θ-estimates, were generally smaller in the south than in the north of 30°S, except for *S. striatus* (Table 7; Fig. 5B). Estimated divergence times varied among taxa. The analyses recovered a most likely divergence time for each of the four taxa analyzed, yet 95% HPD confidence intervals were broad, except for *T. atra*. This is also the species for which estimated divergence time was the most recent (Fig. 5C), confirming that the 30°S phylogeographic break is a historic signature. A common pattern was observed in two of the four species, which had the lowest genetic differentiation of the species analyzed with IMa2, *S. striatus* and *T. atra*. For both species the model predicted asymmetric migration from south to north (and for *T. atra* low but significant migration from north to south) and divergence times were >0, indicating that not all shared haplotypes are a consequence of gene flow but there is also ancestral polymorphism (Fig. 5D; Table 7). As opposed to the other species, the 95% highest posterior density (HPD) of Θ from the north and south do not overlap in *S. striatus* and *T. atra* (Table 7). Altogether the results for these two species do not allow pinpointing a unique cause for the genetic differentiation and the shared alleles among biogeographic regions. The two most differentiated species, *O. tuberculata* and *S. scurra,* showed different results. For *O. tuberculata* migration rates were not significantly differentiated from zero and the degree of differentiation seems best explained by the old splitting (Fig. 5D). *Scurria scurra* does not have significant migration and estimated divergence time is an order of magnitude more recent than for *O. tuberculata* (Table 7). Given that there was no significant inferred migration, the shared haplotypes between biogeographic regions for *S. scurra* are likely due to shared ancestral polymorphism. *Scurra scurra* had the lowest inferred Ne, with Θ-values of 2.08 and 0.81 for the north and south respectively.

**Table 7:** Estimates of migration rates (*m*) in each direction across 30°S, time of divergence (*t*) between biogeographic regions, and genetic diversities (Θ) (north = Θ_N_; south = Θ_S_; ancestral = Θ_A_), based on the isolation-with-migration model implemented in IMa2.

Species		*m*_N→S_	*m*_S→N_	*t* 2% (Kyr)	*t* 10% (Kyr)	Θ_N_	Θ_S_	Θ_A_
*Orchestoidea tuberculata*	HP	0.00	0.00	548.90	109.80	14.66	4.96	29.21
	95% HPD	0.00–0.17	0.00–0.33	335.70–1,665	67.10–333	6.64–29.84	0.00–196.50	15.50–56.60
*Scurria scurra*	HP	0.419	0.01	50.70	10.20	2.08	0.81	5.79
	95% HPD	0–6.32	0.00–5.76	14.90–699.40	3.00–139.90	0.55–7.60	0.18–44.07	2.15–15.62
*Stichaster striatus*	HP	0.08	0.73*	83.30	16.70	21	1,703	47.00
	95% HPD	0.00–0.76	0.19–1.77*	53.40–233.20	10.70–46.60	3–53	771–1,999	23.00–85.00
*Tegula atra*	HP	0.25**	0.73***	15.50	3.10	169.50	52.40	151.50
	HPD 95%	0.05–0.71**	0.33–1.34***	7.50–33.20	1.50–6.60	106.50–310.50	42–68.40	94.50–280.50

For each parameter per species, the high point (HP) and 95% highest posterior density (95% HPD) of the marginal posterior probabilities are shown. Significant m values of the LRT are denoted with asterisks; *P<0.05, **P<0.01, ***P<0.001. *t* was scaled using substitution rates of 2% and 10% per million years, *t* 2% and *t* 10% respectively as is expressed in thousands of years (Kyr).

Significant values are in bold.

## Discussion

### Reinforcing the Marine Connectivity Paradigm

Our results suggest that the phylogeographic patterns of marine benthic organisms of the Chilean intertidal have been strongly influenced by their dispersal potential. Compared to other regions of the world, such as the northeast Pacific [8], southeast Australian coast [12], southeast Africa [41], Hawaii [42], for the southeast Pacific coast under the influence of the Humboldt Current, dispersal potential was significantly associated to genetic diversity, genetic differentiation, and the concordance between phylogeographic and biogeographic breaks, adjusting to the predictions of the marine connectivity paradigm. Four of the five species categorized with high dispersal potential had low genetic differentiation, high genetic diversity at a macrogeographic scale and lacked phylogeographic discontinuities. In contrast, brooders and species with short-lived planktonic larvae had a strong phylogeographic discontinuity at 30°S coincident with the biogeographic break for marine benthic species [31]–[32].

Several studies have explored the determinants of the geographic structure of the genetic diversity of marine species. Among them, Weersing & Toonen [6] tested the general connectivity paradigm, but contrary to the results obtained herein, when including and excluding the brooder from their analyses, they found no support for the general predictions of the marine connectivity paradigm. They found that when excluding brooders, dispersal potential was not correlated with genetic differentiation and concluded that among taxa with free-living larvae, dispersal potential does not predict genetic structure. When we eliminated the one brooder for analyses, differences between contrasting dispersal potentials continued to be significant, albeit lower statistical power when eliminating one of the few low dispersers. In the study of Weersing & Toonen [6], variance between genetic markers explained most of the genetic variability detected, rather than dispersal potential. This is an expected result when markers analyzed between species differ both in quantity and quality of information. In our study, the same portion of the mitochondrial COI gene was used for analyzing all species, reducing the effect of marker choice from the interpretation of results. Similarly, Kelly & Palumbi [8] found that when considering only species with pelagic larvae for analyses, dispersal potential was not a predictor of population genetic differentiation in the northeast Pacific, and that, in some cases, there was a correlation between habitat (high to low intertidal) and genetic differentiation. Likewise, Ayre *et al.* [12] determined that habitat explained the presence of a biogeographic break for the southwest Australian coast better than dispersal potential. To avoid this bias, we analyzed species inhabiting a similar bathymetric stretch and substrata. The exceptions are *E. analoga* and *O. tuberculata* that inhabit sandy beaches. All the rest are all found in the mid-intertidal rocky shore. The species with the most divergent habitat is *O. tuberculata* that inhabits the supralittoral (Table 1), however, eliminating it from the analyses did not change the contrasting phylogeographic patterns between high and low dispersers. Moreover, the studied region is relatively homogeneous in terms of environmental conditions, with the major influence of the Humboldt Current system that maintains upwelling conditions between 42°S and 6°S [31], therefore reducing the potential environmental bias likely occurring in studies covering long stretches of coast (more than 2,600 km). Recently Dawson [43] showed a strong correlation between dispersal potential and genetic differentiation at a regional scale when analyzing co-distributed species. Therefore, when sources of errors are avoided (i.e., same marker and sampling scheme of co-distributed species to enable comparative analyzes), PLD seems to reflect scales of dispersal on the basis of its good fit with genetic differentiation (*F_ST_*) [10]. The phylogeographic patterns detected herein are robust in spite of having used a single mitochondrial marker. There was a consistent association between dispersal potential and genetic structure, suggesting that dispersal potential is a strong determinant of genetic diversity, genetic differentiation, and presence of a genetic discontinuity coincident with a biogeographic transition zone of coastal benthic species along the HCS. Additionally for genetic differentiation the significant interaction of dispersal with the differentiation between biogeographic regions suggests that dispersal alone cannot explain the detected pattern of genetic differentiation, and that the break at 30°S has an important effect.

One species, *S. striatus*, was assumed to have high dispersal potential based on the estimated PLD of its congener *S. australis* [44]. This species did not show a consistent pattern with the other four high dispersers analyzed, yet it is not as structured as the three species considered as low dispersers. Analyzes excluding *S. striatus* did not change the implications of results, i.e. dispersal potential was still a strong predictor of genetic diversity, genetic differentiation, and of the presence of a phylogeographic break at 30°S. Of the four species with structure across 30°S, *S. striatus* had the least structure. For example, Φ*_CT_* (among groups) was non-significant for *S. striatus* while it was for all low dispersers. When analyzing genetic differentiation and isolation by distance of *S. striatus* at each side of the break, only the northern biogeographic region had significant differentiation and isolation by distance. The southern region showed non-significant differentiation and lack of an isolation by distance pattern. Contrastingly, all low dispersers had significant genetic differentiation and isolation by distance in both biogeographic regions. There was a tremendous difference between the genetic diversity (2 Ne) of the northern and southern biogeographic regions for *S. striatus.* Presumably the genus *Stichaster* has cold-water affinities [45] and has colonized the Chilean coast from south to north in the presence of a biogeographic barrier that has nonetheless allowed significant migration. Until its larval duration is properly studied, there are at least two plausible explanations to the detected patterns in *S. striatus*: (1) *S. striatus* is a true high disperser and its spatial genetic structure is the result of the effects of strong local genetic drift, or (2) it is a medium to low disperser, in which case observed results would be consistent with the marine connectivity paradigm. The effective size inferred from isolation-with-migration model is comparable to the two most structured species (*O. tuberculata* and *S. scurra*) and much smaller than the other low dispersal species, *T. atra*, suggesting that genetic drift may indeed have been strong in *S. striatus*. On the other hand, PLD is highly variable within the order Forcipulatidae, with a minimum of zero days (e.g. [46]–[48]), an intermediate value of 80 days [49] and a maximum of 150 days [50]. Larval development of forcipulatids may be abbreviated in response to feeding, habitat [51]–[52], and temperature [49], [53]–[54]. Settlement can be selective, in which case metamorphosis is avoided until the adequate substrate or settling signal is encountered [44], [53], [55]–[56]. On the other hand, the highly productive Humboldt Current System that includes several upwelling regions [57] could favor the fast settlement and metamorphosis of larvae by providing the adequate environmental cues [58]–[59]. Given the high variability of PLD within Forcipulatidae, and the results obtained herein, it is likely that *S. striatus* has a shorter PLD than assigned based on the congener’s planktonic larval duration. For *S. scurra* and *T. atra,* PLD was also assumed based on related species, however, PLD’s are conserved for these gastropod groups. For confamilials of the genus *Scurra* the minimum PLD is 3 days [60] and the maximum 7 days [61], while among the genus *Tegula*, the minimum PLD is 4 days and the maximum 7 days [62]–[63].

There is an interesting contrast between our results and those of Kelly & Palumbi [8] for the genus *Tegula*, probably due to the contrasting sampling scale. We found significant genetic structure in *T. atra* along 21 degrees of latitude while the congener *T. funebralis* from California did not show structure in 8 degrees analyzed [8]. We performed exploratory analyses of the COI data of *T. atra*, considering distances of ca. 8 latitudinal degrees, to better match the spatial scale considered for *T. funebralis*. If sub-samples were drawn from the area north of 30°S, without crossing the 30°S biogeographic break, genetic differentiation was zero and non-significant, mimicking the lack of structure detected for *T. funebralis* in California. As was emphasized by Pelc *et al.* [26] in a meta-analysis, it is very important to consider the spatial scale at which processes such as phylogeographic discontinuities and genetic differentiation may occur with respect to dispersal potential.

### Indirect Consequences of Reduced Dispersal on Genetic Diversity and Divergence

Species with low dispersal potential had consistently and significantly lower genetic diversity (i.e., the lowest values for haplotype and nucleotide diversity). The main way in which populations and species loose genetic diversity at neutral markers is by genetic drift. However, the actual influence of genetic drift depends on its interaction with other evolutionary forces, such as gene flow: reduced dispersal may allow local populations to demographically fluctuate and increase extinction-recolonization rates [64]. Therefore, at a same population density, high gene flow species will retain more alleles than low gene flow species. Consequently, dispersal potential is a major determinant of genetic diversity when comparing species with similar population densities. In addition, population dynamics may also have a strong influence on the effective population size, and therefore on the accumulation of new alleles. For instance, demographic dynamics differ between brooders and broadcasters leading to disparate changes in nucleotide substitution rates [65]–[67]. The accelerated molecular evolution of low dispersers, as a consequence of greater population turnover at various temporal scales can thus also partly explain the contrasting evolutionary patterns between high and low dispersers.

Finally, because low dispersers have subdivided populations they are prone to metapopulation dynamics, i.e. extinction and recolonization [64]. Extinction rates, as well as speciation rates are high in the geologic record of low dispersers [68]–[72]. These processes are likely reinforcing the role of drift and contribute to explaining why low dispersers are more susceptible to accumulate genetic divergence between local lineages, and overall retain less genetic diversity through time than high dispersers.

### The 30°S Biogeographic Break in the Coast of Chile is a Historic Discontinuity

Bayesian analyses based on the isolation-with-migration model take full advantage of the data by using a coalescent approach to jointly estimate migration, time of divergence and effective population size. Ideally, when using coalescent approaches such as those implemented in IMa2, several polymorphic and unlinked loci should be used for a robust estimation of parameters and to deal with phylogenetic uncertainty [73]–[77]. Herein, albeit using a single locus, isolation-with-migration analyses shed important light into the historic nature of the phylogeographic break. Of the four species with a phylogeographic break at ca. 30°S, three species (*S. scurra*, *T. atra* and *S. striatus*) showed similar and relatively recent divergence times, suggesting that the genetic discontinuity originated either during the last glacial period (based on *t* 2%) or just after (when based in *t* 10%). However, *O. tuberculata* had a divergence time between biogeographic regions of an order of magnitude higher and overlaps those estimated for other reported genetic discontinuities in the same region, such as the kelp *Lessonia nigrescens* with estimated divergence at 3095 ky ±1200 ky [78]. These results strongly suggest that the 30°S biogeographic break has an ancient origin. The biogeographic break likely disrupted connectivity between biogeographic regions in the past, rather than in present days, with low dispersers retaining genetic signatures that are geographically concordant with the biogeographic break, while those signatures have been erased in high dispersers by gene flow across the 30° region.

Migration estimations suggested low yet significant northward migration across the 30°S break in two of the four species that had genetic structure among biogeographic regions, *S. striatus* and *T. atra*. Both species showed a greater contribution from the southern to the northern region. Likely these two species have been able to achieve a low yet significant connectivity between biogeographic regions (probably enhanced by the Humboldt Current), slightly eroding the historic signature, which is anyhow strong in both species. Concordantly, shared haplotypes of *S. striatus* and *T. atra* between biogeographic regions are best explained by a combination of migration, shared ancestral polymorphism and drift. For the northeast Pacific coast, Wares *et al.* [28] described a similar scenario of migration in the direction of the main currents and across the Point Conception biogeographic break, for four benthic species with planktonic development. Similarly, along the northern Chilean coast (25°–28°S), asymmetric dispersal linked to the direction of the Humboldt Current has been detected for one non-neutral microsatellite locus of the mussel *Perumytilus purpuratus* [79], and with RAPD data for the intertidal algae *Mazzaella laminarioides* [80].

Consistently dispersal potential was the variable that always best explained the contrasting genetic structure of low and high dispersers. Genetic structuring measures that are more associated with genetic divergence (i.e., nucleotide diversity and genetic differentiation), not only were determined by dispersal potential but also significantly by its interaction with biogeographic region. Based on isolation-with-migration analyses, the biogeographic regions have been completely or partially isolated in recent times. Isolation has led to differentiation, which in some cases is likely to have been enhanced by low effective population size. Genetic diversity was estimated to be unequal between biogeographic regions and species, such that some species (such as *O. tuberculata* and *S. scurra*) and biogeographic regions (the north for *S. striatus* and the south for *T. atra*) may have stronger effects of genetic drift. Concomitantly, biogeographic regions carry within their structure the signatures of historic effective population size, low migration and historic isolation, which probably leads to the significant interaction of dispersal potential and biogeographic region in models associated to nucleotide diversity and genetic differentiation between low and high dispersers.

The current study contributes significantly to the knowledge of COI phylogeography of benthic marine species along the 30°S biogeographic transition zone on the coast of Chile. Previous studies had shown that there was a strong association between the spatial structure of the genetic diversity around 30°S and dispersal potential. Tellier *et al.* [33] and Montecinos *et al.* [34], found sharp genetic discontinuities at 30°S the algae *Lessonia* spp. and *Mazzaella laminarioides*, respectively, evidencing cryptic species at each side of the break. In the former, there is also evidence of reproductive isolation in natural populations [81]. Likewise, studies on animal species also show the consistency of the pattern. The low dispersing gastropods *Acanthina monodon* and *Crepipatella dilatata* have a phylogeographic discontinuity at 30°S [35], [82]. Moreover, there is evidence of gene flow restrictions in the barnacle *Notochthamalus scabrosus* around 30°S, in particular of the two haplogroups present, one has very low abundance north of 30°S [83]. These examples added to the three low dispersers reported herein that have a coincident phylogeographic discontinuity, and the lack of such phylogeographic break in most high dispersal species [36]–[38], [83], give strong support to consistency of the pattern and the validity of the marine connectivity paradigm for the biogeographic break at 30°S on the coast of Chile.

### Concluding Remarks

When designing comparative phylogeographic analyses it should not be assumed, as much of the literature has suggested recently, that compared to other factors, dispersal potential is a weak explanatory variable for spatial genetic structure at macrogeographic scales. Herein we showed that along the coast of Chile under the influence of the Humboldt Current, there is concordance between phylogeographic and biogeographic discontinuities on low dispersing taxa and that these taxa have significantly lower genetic diversity and greater genetic differentiation than species with long planktonic larval duration. Thus, dispersal potential is a robust predictor of the genetic structure of benthic species for the coastline analyzed. This result calls for caution with generalizations as to which are the main factors structuring genetic diversity, and to not extrapolate from studies performed in other regions. This comparative study of species along ca. 2,600 km and other studies of benthic species along the central-northern coast of Chile, support the conclusion that on the coast of Chile, the general marine connectivity paradigm, based on dispersal potential, is met and that phylogeographic patterns could be used as a proxy of dispersal capacity and vice versa.

## Materials and Methods

### Estimation of the Dispersal Potential of Model Species

Amphipods, as all peracarids, brood their embryos up to a crawl-away stage. Thus, *O. tuberculata* has zero days of PLD. Both gastropods analyzed, *S. scurra* and *T. atra*, theoretically have relatively short-lived larvae. Since there are no direct estimations of larval duration of these species, the larval durations considered herein are based on reported larval durations of taxa phylogenetically close to them. For *S. scurra* the estimated PLD considered (7 to 10 days) is based on reports that show that PLD is short and very conserved in the Patellogastropoda [84], [60]–[61]. Similarly, species of the genus *Tegula* have conserved PLD’s of 6 to 7 days [63], [85], which was herein considered as the dispersal potential for *T. atra*.

Of the analyzed species, the ones with longest planktonic larval duration of over 90 days are *E. analoga* [86] and *T. niger* [87]. Unpublished data (S. Navarrete, personal communication) indicates that under laboratory conditions, larvae of *H. helianthus* can persist for at least 2 months without reaching metamorphosis. The New Zealand starfish *Stichaster australis,* the only congener of *S. striatus* from the southeast Pacific coast, has larvae that spend 38 days in the water column [52], [55]. This larval duration was used as for *S. striatus*; albeit it is possible this extrapolation is wrong given that PLD is highly variable in asteroids (e.g. [48]–[50]). Larval development of *Petrolisthes violaceus* lasts 25 days under laboratory conditions at 19°C [88], akin to what has been observed for other members of the genus at similar temperatures [89].

### Data Collection

Individuals of each species were collected from the intertidal zone from Arica (18°S) to Chiloé (42°S) along the coast of Chile. The intertidal areas are not privately owned in Chile, however, there are areas of restricted access (management or protected areas) where we did not sample. All sampling was performed in free access areas for which no permit was required. Also no permits were required for the sampling as species studied did not involve commercially important species or species endangered or protected.

Organisms were processed a few hours after being extracted from nature, obtaining from them a piece of tissue. In most cases tissues corresponded to skeletal muscle. From starfishes (*H. helianthus* and *S. striatus*) and the urchin (*T. niger*), ambulacral feet and gonad tissue were sampled, respectively. Tissues were placed in absolute ethanol and stored at −20°C. Total DNA was extracted using standard phenol/chloroform protocol [90].

A partial sequence of COI was obtained using the universal HCO and LCO primers [91] for *O. tuberculata*, *S. scurra*, and *T. atra*. For other taxa specific primers were used to amplify COI gene: for starfishes primers COIceR and COIceF [92]; for *E. analoga* primers ECL1-ECH1 [93]; for *Petrolisthes violaceus* primers COif [94] and COIa1 [95]; and for the urchin the *de novo* designed primers ArbaF (5′ TTTCTACAAACCATAAGGATATTGG′3) and ArbaR (5′TATACTTCAGGGTGTCCAAAGAATCA′3). These latter primers were designed for this study in GENEIOUS 5.5.4 [96] using COI sequences from other echinoids obtained from GenBank as references. PCR’s were performed in a total volume of 10 µl, which included 1 µl of total genomic DNA, 1.5 mM MgCl_2_, 0.4 µM of each primer, 0.22 mM of each dNTP, 1.5 mg ml^−1^ of bovine serum albumin, and 1.5 U of Taq polymerase. Cycling conditions consisted of an initial denaturation at 94°C for 10 min followed by 35 cycles of 1 min denaturing, 1 min annealing at 50°C, and a 2 min extension at 72°C.

### Data Analyses

For each species, sequences were aligned and then truncated on both extremes using GENEIOUS. DnaSP 5.0 [97] was used for the estimation of per population and global haplotype and nucleotide diversities, as well as the S_nn_ statistic [98] and its significance that was evaluated with 1000 permutations. S_nn_ or the nearest neighbor statistic [98] is a genetic distance measure based on how frequently the nearest haplotypes are in the same sampling locality. It is particularly suitable when data shows high diversity and small or unequal sample size between sites or regions; it outperforms other measures when sample size is small.

Pairwise and global population genetic differentiation values based on sequence data (Φ_ST_) were calculated in ARLEQUIN 3.5 [99]. ARLEQUIN was also used for testing for Isolation by Distance using a Mantel test with 1000 permutations, to determine the correlation between the Slatkin’s linearized population pairwise genetic differentiation [100] and the lineal geographic distance (Km) between populations. Pairwise linear distance matrixes were calculated using the package FOSSIL in R 2.14.0 [101]. Tests for Isolation by Distance were also performed separately north and south of 30°S for species that displayed significant S_nn_ and Φ_ST_.

To test the influence of dispersal capacity and the 30°S transition zone on the genetic diversity and differentiation Generalized Lineal Mixed Models (GLMMs) were performed using the package lme4 in R. These models are appropriate for data influenced both fixed and random effects. In the models population-specific data of genetic diversity were grouped into biogeographic regions (north and south of 30°S). For models of genetic differentiation data was grouped according to biogeographic differentiation (Φ_ST_ among and within regions). All models were constructed nesting the species as a random factor. The syntax of the diversity models was: Genetic Diversity = Dispersal X Biogeographic Region+Species (Dispersal). For the differentiation model the syntax was: Genetic Differentiation = Dispersal X Biogeographic Differentiation+Species (Dispersal).

Haplotype networks for each species were constructed using NETWORK 4.6 (Fluxus Technology 2010). To evaluate the presence of a genetic discontinuity coincident with the biogeographic break at 30°S, ARLEQUIN software was used to perform AMOVA by grouping individuals *a priori* north and south of 30°S. The difference between the percentages of the genetic variation explained by groups, populations within groups or within populations, between high and low dispersers were evaluated with GLMMs.

Two further analyses were conducted for species that displayed genetic structure with AMOVA, i.e., those that had less than 95% of the variation within populations. The clustering method implemented by SAMOVA 1.0 [102] was executed to define the most likely number of groups of populations, based on the proportion of the total molecular variance explained by differences between groups (F*_CT_*). The number of groups was denoted as *k*. In each species, statistical variance was evaluated with 1000 random permutations running repeatedly SAMOVA from 2 to 1-*n* populations, where *n* was the total number of sampled localities. The *k* value that maximized F*_CT_* was interpreted as the most likely geographic subdivision. A genetic break coincident with the biogeographic transition was inferred if when *k* = 2 the clustering F*_CT_* was significant and the groups of individuals were located north and south of 30°S.

The second analytical approach applied only on species with structure across 30°S, consisted in the evaluation of asymmetric migration and time of divergence across 30°S using the isolation-with-migration demographic model [103] in a Bayesian context implemented in the software IMa2 [73]. This model does not assume equilibrium conditions for the populations and allows to estimates demographic parameters in recently separated populations that share haplotypes as consequence of gen flow or incomplete lineage sorting [74]. The estimated demographic parameters are *t* (splitting time), effective population sizes (Θ** = **2 Ne), and migration rates in each direction across the biogeographic break. For each analyzed species, we generated a random balanced subsample of individuals per biogeographic region with a double goal of having equal number of individuals per biogeographic region and reducing the number of sequences analyzed, which is recommended when using the model of isolation-with-migration with information from a single locus [73]. To estimate the best set of priors that ensure mixing and convergence of parameters we carried out several preliminary runs in the M mode (MCMC mode) of IMa2. All runs were carried out using the HKY model of substitution. Chain length ranged from 50,000,000 to 150,000,000 generations until convergence was achieved. Chains were sampled every 100 generations with the first 10 to 30% discarded as burn-in. When convergence could not be reached, long runs with Metropolis Couples MCMC (MC^3^) algorithm [104] were carried out setting 20 chains with geometric heating (a = 0.96, b = 0.9). After achieving convergence in the M-mode, we used the same simulated genealogies in the L-Mode (Load Tree mode) of full isolation-with-migration model, to calculate the log maximum-likelihood and credibility intervals based on the 95% highest posterior density (95% HPD) for each parameter. The significance of migration patterns was estimated using a likelihood ratio test (LTR) [105]. Splitting time parameter was re-scaled into years (t/μ) using two substitution rates, 2 and 10% per My. Even though 2% per My is a high substitution rate for invertebrates, according to the time dependency of molecular evolution [106], rates of substitution at the population level are several times higher than the ones inferred from interspecies analyses [106]–[107], thus we used two rates and obtained a temporal range for the divergence across 30°S.
